# Advances in Research on Childhood‐Obesity Associated With Gestational Diabetes Mellitus

**DOI:** 10.1111/1753-0407.70212

**Published:** 2026-03-23

**Authors:** Wantong Chen, Minghui Xi, Shuai Li, Luyi Li, Danhuai Zhang, Ming Gong, Yueyang Zhao, Na Wu

**Affiliations:** ^1^ Department of Pediatrics Shengjing Hospital of China Medical University Shenyang China; ^2^ Department of Thoracic Surgery Shengjing Hospital of China Medical University Shenyang China; ^3^ Department of Emergency Shengjing Hospital of China Medical University Shenyang China; ^4^ Department of Library Shengjing Hospital of China Medical University Shenyang China

**Keywords:** child health, childhood obesity, gestational diabetes mellitus, offspring, pregnancy

## Abstract

Childhood obesity has emerged as a major global public health concern. Recent reports indicate that the worldwide obesity rate among children and adolescents has climbed to 8.5%. Obesity‐related conditions present serious health risks. The causes of childhood obesity are multifactorial, with growing attention from researchers on the link between maternal gestational diabetes mellitus (GDM) and an elevated risk of obesity in offspring. This association can have lasting effects throughout childhood. Consequently, understanding the causes of childhood obesity and implementing effective preventive and therapeutic measures are crucial for managing and mitigating the condition and its associated health complications. This study aims to thoroughly explore the relationship between maternal GDM and the heightened risk of childhood obesity, examining developmental stages, underlying pathophysiological mechanisms, and contributing factors. By developing and applying targeted management strategies during a child's growth and development, the objective is to effectively curb and improve obesity outcomes among children born to mothers with GDM.

## Highlights

- In this review, showing a significant correlation between the child‐hood obesity and gestational diabetes mellitus and elucidated its mechanisms.
- The high‐glucose environment may affect the lipid metabolism of their offspring by altering the expression of leptin genes, the activity of lipases, and the concentrations of insulin and C‐peptide. This can lead to increased birth weight, thereby increasing the risk of future obesity.
- Breastfeeding and good glycemic control through lifestyle or medication are protective factors for future childhood obesity.

## Graphical abstract

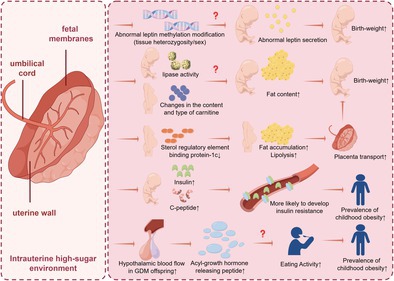

**Abbreviations**
BF: blood flow
BMI: body mass index
BMI‐SD: body mass index standard deviation score
DASH: dietary approaches to stop hypertension
DOHaD: Developmental origins of health and disease
GDM: Gestational diabetes mellitus
GLUT1: Glucose transporter 1
GUTS: Growing up today study
HAPOFUS: hyperglycemia and adverse pregnancy outcomes follow‐up study
hPL: human placental prolactinogen
LDL‐C: low‐density lipoprotein cholesterol
LGA: large‐for‐gestational‐age
LPL: lipoprotein lipase
PG: plasma glucose
TC: total cholesterol
TG: triglyceride
WHO: world health organization
WOF: world obesity federation

## 1 Introduction

Childhood‐obesity has emerged as a critical public health issue in the 21st century. According to the World Health Organization (WHO) 2006 standards for child growth and development, infants and children under 2 years of age are diagnosed using “weight‐for‐length.” This is measured by calculating a standard deviation (or *Z*‐score) based on parameters from a normal population of the same age, sex, and length. A *Z*‐score > +2 standard deviations from the mean weight of the reference population is classified as “overweight,” while a *Z*‐score > +3 is classified as “obese.” For children aged 2 years and older, Body Mass Index (BMI) is used as a diagnostic criterion for obesity. A BMI at or above the 95th percentile for a child's age and sex indicates obesity [1].

The World Obesity Federation (WOF) reports that the rising trend of obesity among children and adolescents has yet to stabilize. Projections estimate that the number of obese children aged 5 to 19 will reach 206 million by 2025 and could increase to 254 million by 2030 [2]. The causes of obesity are multifactorial, with genetic factors accounting for 40%–70% of cases [3]. As early as 1983, researchers observed that children born to mothers with gestational diabetes mellitus (GDM) might be at a higher risk of developing obesity [4].

The global prevalence of GDM among pregnant women is steadily increasing. This trend raises an important question: Is there a direct correlation between the rise in GDM and the growing number of obese children? In 2019, researchers analyzed data from over 40 000 pregnancies and their offspring, covering births between 1995 and 2004. They found that offspring exposed to elevated maternal blood glucose levels during pregnancy (levels not high enough to diagnose GDM) had a 13% increased risk of developing obesity between the ages of 5 and 7. If the mother was diagnosed with GDM, the offspring's risk of childhood obesity increased by 52%. Estimates indicate that in 2017, ~21.3 million live births were affected by some form of gestational hyperglycemia, with GDM accounting for around 18.4 million cases, or 86.4% of all pregnancy‐related hyperglycemia [5].

The Developmental Origins of Health and Disease (DOHaD) theory, proposed in 1995 by David Barker, an epidemiologist at the University of Southampton, U.K., may explain this phenomenon. DOHaD suggests that an individual's health is shaped not only by genetic and lifestyle factors in adulthood but also by environmental influences during early development. The DOHaD theory posits that an individual's health is shaped not only by genetic factors and adult lifestyle but also by environmental influences during early development. Specifically, the theory highlights the critical role of nutritional and environmental conditions in fetal and early infancy stages on long‐term health outcomes.

**FIGURE 1:**
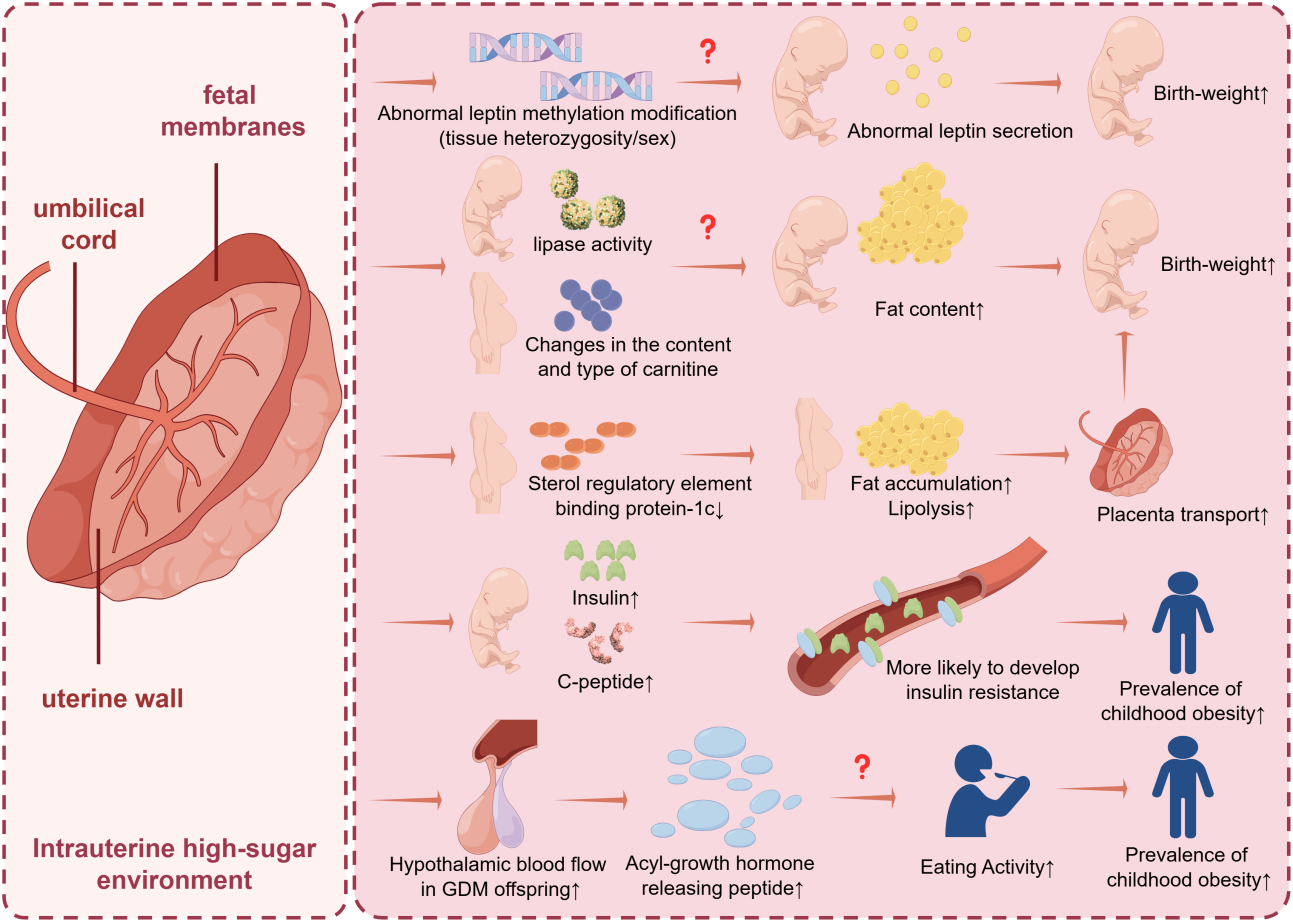
The potential mechanisms of childhood obesity related to gestational diabetes mellitus.

Adverse factors during these early periods, such as maternal illness, can increase the likelihood of developing chronic diseases, including obesity and type 2 diabetes, in later life [6]. For example, GDM (gestational diabetes mellitus) exposes offspring to a high‐glucose environment, potentially affecting fetal growth and development through the placenta and other pathways (Figure 1). Despite ongoing research, the mechanisms linking GDM to childhood obesity remain unclear [7], which complicates efforts to prevent and address this issue effectively.

This review will explore the prevalence of childhood obesity in GDM offspring across various developmental stages (Table 1) and summarize relevant mechanisms and contributing factors, aiming to offer new perspectives for the prevention and treatment of obesity in GDM offspring.

**TABLE 1:** Articles on childhood obesity related to gestational diabetes mellitus.

Year	Country	Type of study	Age (year)	Objective	Outcomes	Quote
Related						
1983	USA	Longitudinal cohort study	0.5–19	218	Obesity in the offspring was directly related to maternal diabetes, and GDM has a significant effect.	[4]
1997	Korean	Population base cohort	0	1935	Increased prevalence of macrosomia with GDM in Korean.	[8]
2009	Poland	Population base cohort	4–9	214	Children born to mothers with gestational diabetes seem to be at risk for obesity and metabolic disturbances.	[9]
2010	Germany	Prospective cohort study	2,8,11	378	The research shows that prevalence of overweight and, as a consequence, HOMA‐IR during childhood is higher in OGDM than in OT1D, indicating that maternal diabetes type affects overweight risk.	[10]
2015	China	Retrospective cohort study	0–5	1263	Offspring born to GDM mothers with pre‐pregnancy overweight/obesity or excessive GWG were associated with increased risks of large for gestational age and macrosomia at birth, and childhood overweight at 1–5 years old.	[11]
2016	France	Retrospective study	0	15 551	GDM was associated with more LGA babies.	[12]
2016	China	Population base cohort	9–11	4740	GDM is associated with an increased risk of obesity in children aged 9–11 years old.	[13]
2017	UK	Population base cohort	0–1	24 000	GDM offspring have increased fat content.	[14]
2017	USA	Prospective cohort study	9–14	15 009	Offspring who were born to a GDM pregnancy were more likely to have high birthweight, male participants who were born to a GDM pregnancy had higher BMI.	[15]
2018	France	Population base cohort	0	1484	GDM is significantly related to increased levels of LGA and fetal macrosomia, independently of obesity.	[16]
2019	Sweden	Prospective cohort	0–1	135	Both maternal glycaemia and obesity are determinants of increased early life adiposity, especially in girls, with glycaemic levels being more influential than maternal weight for infants born to women with GDM.	[17]
2020	Turkey	Population base cohort	2–3	533	GDM increases the incidence of obesity in offspring during the toddler period, especially at the ages of 2 and 3.	[18]
2020	China	Prospective cohort study	1–4	10 412	GDM increases the incidence of obesity in offspring aged 1–4 years old.	[19]
2021	China	Prospective cohort study	0–1	1290	GDM increases the incidence of obesity in offspring during infancy, especially at 1 month and 3 months.	[20]
2022	China	Retrospective cohort study	0	17 260	The association of pre‐pregnancy overweight and obesity with LGA was found to be partially mediated by GDM.	[21]
2022	UK	Prospective cohort study	0	867	Neonatal obesity due to GDM may be associated with altered maternal plasma lipid abundance.	[22]
2022	Korea	Population base cohort	3–5	5276	GDM increases the incidence of obesity in offspring during the preschool period, especially at the ages of 3, 4, and 5.	[23]
2023	Aurora	Observational study	5	727	There was a higher risk of high FM% among offspring of the IR‐hyperglycemic and dyslipidemic‐high FFA subgroups; this risk was of greater magnitude compared with pre‐pregnancy obesity alone, GDM alone, or both conditions.	[24]
2024	Denmark	Longitudinal cohort study	5–9	6794	Pregnancies affected by GDM had higher fetal growth rate and the offspring had a higher risk of being overweight at 5–9 years.	[25]
2024	China	Longitudinal cohort study	6–10	1156	GDM increases the incidence of obesity in offspring during school age, especially at 5.9 years and 8.3 years old.	[26]
2024	USA	Population base cohort	2–6	10 335	GDM increases the incidence of obesity in offspring during school age, especially at the age of 6.	[27]
Unrelated						
1998	USA	Retrospective cohort study	8–10	524	Prenatal exposure to the metabolic effects of mild, diet‐treated GDM does not increase the risk of childhood obesity.	[28]
2013	USA	Population base cohort	2–4	2093	GDM is not related to offspring obesity.	[29]
2010	USA	Population base cohort	2	1165	Childhood obesity at 2 years of age in GDM offspring not associated with maternal GDM.	[30]
2020	USA	Follow‐up study	5–10	716	The relationship between GDM‐associated childhood obesity was not apparent at about 7 years of age.	[31]
2020	Malaysia	Prospective cohort study	0	507	Maternal lipaemia and GWG at a low threshold (> 10 kg) adversely impact neonatal adiposity in Asian offspring, independent of glucose, insulin resistance and pre‐gravid BMI.	[32]

## 2 GDM and the Development of Childhood‐Obesity

### 2.1 Infancy (0–1)

Research has shown that offspring of mothers with GDM exhibit higher intrauterine fetal growth rates, which may contribute to increased body fat and birth weight at birth [33]. Elevated birth weight is an early indicator of risk factors associated with childhood overweight and obesity [34]. Evidence indicates that a non‐fasting blood glucose level exceeding 120 mg/dL during early pregnancy in GDM mothers is linked to a 24% higher risk of macrosomia. Similarly, a glycated hemoglobin level above 44 nmol fructose per 10 g of hemoglobin (representing the 95th percentile of the normal control population) in early pregnancy raises the risk of macrosomia by 25%. In late pregnancy, a non‐fasting blood glucose level over 120 mg/dL increases the risk by 23%, while a glycosylated hemoglobin level greater than 44 nmol fructose per 10 g of hemoglobin elevates the risk to 32% [35].

The occurrence of macrosomia can be explained by the Pedersen hypothesis, which posits that maternal hyperglycemia leads to fetal hyperinsulinemia. This state enhances glucose utilization and fetal fat accumulation [36]. Glucose can cross the placenta when maternal glycemic control is poor, but maternal or exogenous insulin cannot. By mid‐gestation, the fetal pancreas starts secreting insulin, responding to the mother's hyperglycemic environment. The resulting combination of hyperinsulinemia and hyperglycemia drives increased synthesis of fetal fat and protein, raising the risk of macrosomia. This, in turn, may increase the likelihood of childhood obesity in offspring born to mothers with GDM. The intrauterine hyperglycemic environment in GDM widens the glucose concentration gradient between the mother and fetus, leading to excessive glucose transfer from the placenta to the fetus. This, in turn, affects fetal insulin secretion, adipocyte growth, and triglyceride synthesis, ultimately resulting in fetal overgrowth [37]. Glucose Transporter 1 (GLUT1) and Glucose Transporter 3 (GLUT3) are the primary placental glucose transporters in humans, widely expressed during early pregnancy and at term [38]. Mouse studies confirm that compared with normal pregnancies, GDM mice exhibit significantly downregulated phosphorylation levels of Adenosine Monophosphate‐activated Protein Kinase (AMPK) in placental tissue. AMPK in the trophoblast may directly interact with GLUT3, promoting its translocation from the cytoplasm to the cell membrane to mediate glucose transport. In the hyperglycemic environment of GDM, suppressed AMPK activity leads to abnormal GLUT3 localization. This impairs the placenta's intrinsic glucose metabolism, increases net glucose transport from maternal to fetal circulation, and ultimately affects fetal‐placental growth [39].

Currently, most studies have linked GDM with an increased risk of obesity in offspring during infancy. Evidence has suggested that this link is most pronounced at birth and may persist up to 1 month and even 3 months of age [20]. A 1997 study indicated that mothers with GDM had a higher risk of giving birth to macrosomic infants, with findings showing elevated umbilical cord serum concentrations of C‐peptide and insulin, both strongly correlated with birth weight [8]. Even after adjusting for multiple risk factors, research has demonstrated that newborns of GDM mothers are at an increased risk of adverse neonatal outcomes, with the risk of macrosomia being up to three times higher compared with the general population [40].

However, some studies have proposed that pre‐pregnancy overweight and obesity in GDM mothers are primary contributors to obesity in their offspring [29]. Similarly, a large prospective study in Spain supported this view but also confirmed that GDM can mediate the relationship between pre‐pregnancy overweight/obesity and high birth weight [21]. Although GDM treatment can normalize birth weight, infants born to GDM mothers tend to experience substantial weight gains between 3 and 12 months of age to compensate for birth weight reduction [41]. This phenomenon may be linked to increased fat content and elevated lipoprotein lipase (LPL) activity [14, 42, 43].

Furthermore, altered lipid metabolic profiles in GDM mothers, indicated by lipidomic assessments, revealed significant differences in lipid species abundance compared with normoglycemic mothers, identifying nine adipogenesis‐related lipid species associated with large‐for‐gestational‐age (LGA) outcomes [22]. Elevated maternal serum triglyceride (TG) levels and lower HDL‐C levels during pregnancy have also been linked to higher neonatal birth weight [44, 45]. Specifically, fasting and 1‐h postprandial TG levels at 16 weeks of gestation have shown strong associations with neonatal obesity [46], and TG levels at delivery have been independently linked to LGA [47]. Despite these findings, the precise mechanisms remain unclear and warrant further investigation.

Further research indicates that GDM can lead to genomic changes in offspring, which are associated with the development of macrosomia. As early as 2008, researchers have noted that obesity in offspring born to mothers with GDM may be linked to abnormal methylation modifications of the imprinted gene MEST [48]. DNA methylation refers to the covalent bonding of a methyl group to the 5′ carbon position of cytosine within the tandem repeat sequence, which consists of cytosine and guanine in the genome, facilitated by DNA methyltransferases. Offspring exposed to intrauterine hyperglycemic environments experience programmed alterations in metabolic patterns, with effects that can persist into adulthood.

Subsequently, a study examining three candidate genes in the placentas of GDM versus non‐GDM pregnant women found that elevated maternal blood glucose levels were associated with hypermethylation on the maternal side and hypomethylation on the fetal side of the leptin gene promoter region in placental tissues. Additionally, hypomethylation was observed in the promoter regions of the lipocalin and lipoproteinase genes [49]. This finding was corroborated by a study conducted by Lesseur et al., which experimentally demonstrated that maternal metabolic status before and during pregnancy alters placental DNA methylation profiles at birth. This alteration leads to elevated placental leptin methylation in infants exposed to gestational diabetes, potentially linking it to metabolic programming for obesity and related diseases [50], the process is shown in Figure 1.

Furthermore, single nucleotide polymorphisms in FAM13A have been associated with BMI and lipid traits [51, 52], underscoring the potential role of their methylation in increasing the risk of chronic metabolic diseases, such as obesity, in future offspring. Additionally, researchers found that mid‐pregnancy carnitine changes in mothers with GDM were linked to the development of macrosomia in their offspring [53], which may heighten the risk of childhood obesity. However, whether changes in carnitine levels in the offspring of GDM mothers are similarly associated with childhood obesity, and whether there is also an association with alterations in other metabolic markers, requires further investigation.

Researchers have found that prolonged breastfeeding can mitigate obesity in infants born to mothers with GDM and serves as a crucial protective factor against future childhood obesity, particularly after 6 months of age [54] and over the subsequent 3 years [55]. The study indicated that the average weight of breastfed infants began to decline between 6 and 8 months, becoming significantly lower than that of formula‐fed infants from 6 to 18 months. This difference was associated with the metabolic effects observed when infants are completely removed from maternal influence.

The intrauterine environment significantly affects fetal growth, suggesting that the placenta may play a role in intrauterine programming related to GDM and childhood obesity [56]. Exposure of the placenta to a high‐glucose environment during GDM pregnancies impacts placental growth [57], particularly in women exhibiting relative insulin resistance, a condition characteristic of GDM [58]. Furthermore, human placental prolactinogen (hPL) promotes insulin resistance, a process exacerbated by the placental secretion of glucocorticoid‐releasing factor, which stimulates glycolysis and fatty acid mobilization, thus worsening insulin resistance [59]. Chronic hyperglycemic stimulation in the GDM placental model results in decreased expression of sterol regulatory element‐binding protein‐1c and increased fat deposition. The accumulated fat can undergo lipolysis, releasing more fat and its metabolites to the fetus, which can lead to conditions such as high birth weight [60], thereby increasing the risk of future obesity in offspring.

### 2.2 Toddlerhood (1–3)

Currently, most studies indicate that GDM is positively associated with an increased prevalence of obesity in offspring during early childhood, with a more pronounced effect observed from the age of 2 years onward. Initially, research on obesity in offspring primarily focused on developed countries. However, as global obesity rates rise and populations age, researchers are increasingly turning their attention to developing countries, particularly regarding younger populations. As research continues to advance, it becomes evident that obesity at a younger age, along with prolonged obesity, significantly impacts the future quality of life for individuals and imposes a greater social burden.

In 2017, Li et al. concluded for the first time from a cohort study that the prevalence of overweight (including obesity) was elevated among offspring of mothers with GDM at 1–3 years of age [55]. Similarly, Shi et al. found that GDM offspring had an increased risk of overweight or obesity at 1–4 years of age [19]. Bider‐Canfield et al., after adjusting for confounders, also concluded that GDM increases the risk of childhood obesity in offspring aged 2 years [61]. In 2020, a large Turkish study adjusted the age of its domestic obesity study population to ≤ 5 years. Collaborating with 45 healthcare organizations, researchers conducted retrospective studies that identified GDM as an independent risk factor for obesity in offspring, particularly for childhood obesity occurring at ages 2 and 3 years [18]. However, it has also been noted that GDM shows little to no association with the onset of obesity in offspring at 2 years of age [30].

Additionally, maternal blood glucose levels during pregnancy have been significantly associated with an increased incidence of macrosomia in offspring, but the positive correlation between maternal blood glucose levels during pregnancy and the risk of overweight in offspring diminishes over the subsequent 3 years [55]. This finding prompted us to consider whether improvements made before conception and during pregnancy, as well as the young child's own growth and development, could mitigate some of the effects of GDM on childhood obesity in early childhood.

The findings may also be related to diabetes care before and during pregnancy, particularly regarding diet and exercise [61]. Statistics on the lifestyles of mothers with GDM, including pre‐pregnancy BMI and appropriate weight gain during pregnancy, indicate that a healthy lifestyle can reduce the incidence of obesity in their offspring between the ages of 1 and 5 years [11]. Details on how lifestyle improvements in GDM mothers will be discussed later. The study also found that the association between childhood overweight and maternal pre‐pregnancy obesity was generally stronger in older children, whereas the link between childhood overweight and excessive gestational weight gain (GWG) tended to be weaker in this age group.

Although offspring of mothers exposed to GDM are at increased risk for neonatal obesity and childhood obesity after age 5, it remains unclear whether the impact of excessive GWG in GDM mothers on their offspring's overweight status diminishes as the children grow older. Therefore, further research is necessary to address this question.

In terms of growth and development characteristics, early childhood is a critical period marked by a peak in growth, characterized by a higher metabolic rate and increased energy demands. Additionally, children in early childhood exhibit greater curiosity about their surroundings and engage in more physical activity. Regarding eating habits, this period is crucial for transitioning to a more varied diet. Parents should pay particular attention to the types and combinations of foods their children consume at each meal and throughout the day. Consequently, it is essential to emphasize lifestyle education for GDM mothers and their children in our clinical practice.

### 2.3 Pre‐School and School Age (3‐)

Researchers have found that elevated rates of preschool obesity are more likely to occur in male offspring [15]. The risk of obesity at 4 years of age can be 1.78 times higher in children whose mothers have GDM compared with those whose mothers do not [62]. A 2019 study identified GDM as an independent risk factor for the development of obesity in offspring aged 1 to 6 years [63]. In 2022, Choi et al. demonstrated that mothers with GDM increased the likelihood of obesity in their children by 1.58 times more than in children of mothers without GDM, particularly by age 5 [23]. Furthermore, a 2024 study by Li et al. noted that maternal GDM was a significant risk factor for overweight or obesity in preschool and school‐age children, particularly at ages 5.9 and 8.3 years, with the association potentially strengthening with age [26]. This risk may also be influenced by the child's gender. Additionally, Boerschmann et al. suggested that GDM is linked to childhood obesity occurring in offspring before the age of 11 years [10].

A prospective cohort study involving 15 009 participants, followed for 14 years in the Growing Up Today Study (GUTS), found that maternal GDM was associated with a higher risk of obesity in late childhood, adolescence, and early adulthood, particularly among male participants [15]. GDM has been linked to an increased incidence of obesity in school‐age offspring. However, our findings indicate that this relationship may diminish during the later school years. This decline could be attributed to the growing time that offspring spend outside the influence of their mother's high‐sugar environment, alongside a gradual reduction in GDM's impact on their metabolism. Nevertheless, this does not imply that GDM is not associated with an elevated risk of obesity in offspring. It may be possible to mitigate some of the effects of GDM through a conscious lifestyle that includes a balanced diet and regular exercise as the offspring mature.

In a 2005 study conducted in India, a positive association was observed between mothers with GDM and their offspring being heavier at 7.7 years of age [64]. Ouyang et al. found that GDM was associated with higher BMI in U.S. offspring from birth to 7 years [56]. Similarly, Le Moullec et al. reported a higher prevalence of obesity in offspring of mothers with GDM compared with those of mothers without GDM at ages 5–7, with a notable prevalence in male offspring [65]. Additionally, research by Lingwood et al. corroborated these findings, revealing significantly higher rates of obesity in males, particularly at age 8 [66]. Furthermore, Catalano et al. not only noted a higher prevalence of obesity in offspring of mothers with GDM compared with those of normal mothers at ages 7–10.6, but also discovered that these offspring exhibited a greater index of insulin resistance [67].

Research suggests that childhood obesity is significantly associated with 10 amino acid metabolites and 20 fatty acid metabolites. These key metabolites, in conjunction with the body mass index standard deviation score (BMI‐SD), can effectively predict the risk of insulin resistance (IR) and may serve as future predictors of childhood obesity. However, further experiments are needed to confirm whether these metabolites are present in GDM [68]. Adipose tissue is a major site of insulin resistance, with the degree of resistance correlating with adipose tissue metabolism; this association is notably stronger in obese individuals [68]. Adipogenesis, the process of expanding white adipose tissue, plays a crucial role in the development of obesity [69].

In 2009, Michelle Colomiere et al. demonstrated post‐receptor defects in the insulin signaling pathway in the placentas of pregnant women with GDM [70], which may contribute to insulin resistance in GDM offspring [10]. During the pathological progression of obesity, white adipose tissue becomes severely dysfunctional and is unable to proliferate adequately to store excess energy, resulting in ectopic fat deposition in other tissues, a phenomenon referred to as “lipotoxicity.” This condition can increase the risk of systemic insulin resistance [71, 72]. Additionally, adipose tissue can exacerbate insulin resistance by releasing fatty acids and cytokines [73, 74]. Insulin resistance can promote fat accumulation and inflammatory responses [75, 76, 77].

Adipose tissue inflammation involves the infiltration of macrophages and other immune cells around adipocytes. These macrophages release various inflammatory factors, including TNF‐α, IL‐1β, and IL‐6, which adversely affect organs and tissues such as the liver and muscles, increasing the burden on the organism and leading to various diseases [78]. The inflammatory response can also activate several signaling pathways, including the NF‐κB pathway. Activation of these pathways further amplifies the inflammatory response, promoting additional immune cell infiltration and cytokine release [79], which exacerbates the inflammation. Adipose inflammation disrupts the growth, development, and function of fat cells, ultimately contributing to obesity [80].

Furthermore, a study on adolescents found a correlation between obesity and increased hypothalamic blood flow (BF) [81]. By collecting anthropometric data from 91 children aged 7 to 11 years, researchers discovered that offspring of mothers with GDM who were exposed to a high‐sugar environment in utero were more likely to experience increased BF [82], leading to a heightened risk of future obesity.

Recent studies have indicated that GDM is associated with a diminished correlation to the development of obesity in offspring after the age of 6 years [27]. Similarly, Landon et al. reached a comparable conclusion, noting that GDM was not linked to body mass index (BMI) around age 7 in American children [31]. A U.S. study that simultaneously assessed birth weight and school‐age obesity in offspring found that maternal GDM and higher birth weight correlated with an increased risk of overweight during adolescence. However, the influence of GDM on offspring obesity seems only partially attributable to its effects on birth weight. Researchers adjusted for maternal BMI and concluded that the future development of childhood‐obesity is more strongly associated with maternal BMI [83].

Additionally, a large study involving participants from 12 countries demonstrated that GDM is associated with obesity in offspring aged 9–11 years. In this study, researchers analyzed children's feeding habits and lifestyles, identifying breastfeeding and moderate physical activity as protective factors against the onset of obesity [13]. Other studies have shown that offspring of mothers with GDM have higher food requirements at age 8 and are more inclined to select high‐sugar and high‐fat foods, increasing their likelihood of being overweight or obese [84]. These findings suggest that school‐age children may attain body image control through mindful management of their diet and exercise.

Currently, most studies support the association between GDM and increased childhood obesity in offspring. However, no research has yet examined the long‐term effects of maternal GDM on offspring growth and development from infancy through childhood, adolescence, and into adulthood, particularly regarding obesity. Further research is needed to confirm these findings, and we will continue to monitor developments in this field.

## 3 Factors Associated with the Influence of GDM Mothers on Childhood‐Obesity

### 3.1 GDM Mother

#### 3.1.1 Impact of Pre‐Pregnancy BMI and Gestational Weight Gain (GWG) in GDM Mothers on Childhood‐Obesity in Their Offspring

Currently, there is a consensus that the pre‐pregnancy BMI of mothers with GDM and abnormal weight gain during pregnancy contributes to an increased incidence of obesity in their offspring. Specifically, a pre‐pregnancy BMI of ≥ 25 kg/m^2^ or excessive weight gain during pregnancy is associated with a higher likelihood of the offspring being large for gestational age (LGA) and developing obesity later in life [12, 85]. Furthermore, GDM mothers with high gestational weight gain (GWG) appear to have a more pronounced effect on male offspring [86].

In 2015, a study first established a positive correlation between pre‐pregnancy overweight/obesity and abnormal GWG in GDM mothers and an increased risk of childhood overweight in their offspring at ages 1 to 5 [11]. A 2019 study conducted in the U.S. revealed that GDM and excessive GWG were more strongly linked to offspring BMI trajectories and obesity risk at age 4, particularly among children whose mothers exhibited both risk factors [87].

More recently, in 2024, researchers found that mothers with GDM who were overweight or obese prior to pregnancy had an elevated risk of their offspring becoming obese between the ages of 5 and 9. This study demonstrated that after week 25 of gestation, women with GDM experienced higher fetal growth rates, which correlated with an increased risk of abnormal weights in their offspring. Notably, when the pre‐pregnancy BMI of these mothers was adjusted at week 28, there was a slight decrease in fetal growth rates and a corresponding reduction in the potential risk of obesity in the offspring [25].

#### 3.1.2 The Effect of Abnormal Blood Glucose on Obesity in Offspring

Researchers have found that obesity in offspring of women with GDM is closely associated with maternal blood glucose levels. In 2019, the Hyperglycemia and Adverse Pregnancy Outcomes Follow‐Up Study (HAPOFUS) concluded that exposure to elevated glucose levels in utero is independently linked to childhood obesity. The study identified a positive correlation between maternal glycemic predictors and childhood obesity outcomes by analyzing data from 4832 children aged 10 to 14 years, who were GDM offspring, along with their mothers, from 10 HAPO centers [88].

Consequently, glycemic control is crucial for patients with GDM, as it not only impacts the health of the pregnant woman but is also significantly associated with the development of obesity in their offspring. The effects of GDM on obesity may commence in the fetal stage and persist into early infancy, elevating the risk of obesity during childhood, adolescence, and even adulthood. Furthermore, it has been suggested that children born to mothers with GDM experience rapid growth during the early postnatal period, particularly within the first year of life, which may considerably heighten the risk of obesity in adulthood [89].

##### 3.1.2.1 The Effect of Glycemic Control With Insulin and Metformin in GDM Mothers on Childhood‐Obesity in Their Offspring

Currently, GDM can be managed through dietary control or medication. Some studies have demonstrated that glycemic control with insulin and metformin in mothers with GDM can reduce the risk of childhood obesity in their offspring, with insulin appearing to be more effective in the long term [28, 90, 91]. Tarry‐Adkins et al. found that newborns of GDM mothers treated with metformin during pregnancy were significantly less likely to be larger than gestational age or to be born macrosomic compared with those of GDM mothers treated with insulin. Although the mean birth weights of infants born to mothers who used metformin were lower, these children exhibited accelerated postnatal growth, leading to heavier weights in infancy and higher BMIs by mid‐childhood compared with children whose mothers received insulin treatment [92].

Similar results were observed in animal studies, where the body weights of offspring from GDM mice were significantly greater than those of offspring from normal mice. The body weights of the GDM offspring improved significantly with treatment using insulin and metformin. Additionally, the study indicated that the use of these two medications during gestation had a long‐term and sustained impact on lipid metabolism in male offspring of GDM mice. This was primarily evidenced by reduced plasma total cholesterol (TC), TGs, and low‐density lipoprotein cholesterol (LDL‐C) levels, along with decreased hepatic lipid content. In contrast, no significant changes were observed in these parameters in female offspring across the different treatment groups [93]. This finding provides a theoretical basis for predicting treatment effects based on the sex of the offspring.

#### 3.1.3 Effect of Lifestyle of GDM Mothers on Childhood‐Obesity in Their Offspring

Most researchers agree that lifestyle management for mothers with GDM effectively reduces the risk of larger‐than‐gestational‐age babies and macrosomia in their offspring [94]. It is recommended that such management begins before pregnancy [95]. The mother's lifestyle significantly impacts the future growth and development of her children, particularly in the areas of diet and exercise. A review by Brown et al. demonstrated that newborns of pregnant women diagnosed with GDM who received lifestyle intervention (e.g., dietary advice, physical activity, education, or self‐monitoring of blood glucose) benefited primarily from reduced fetal growth. Compared with the group without lifestyle interventions, those receiving treatment showed a reduced incidence of macrosomia [96]. While lifestyle management has some effect on the future development of childhood‐obesity in GDM offspring, this is limited, primarily due to the late intervention; changes in maternal and placental function generally occur during the first trimester of pregnancy [97].

##### 3.1.3.1 The Effect of Diet in GDM Mothers on Childhood‐Obesity in Their Offspring

Early studies indicate that GDM can reduce the risk of obesity in future offspring through dietary interventions [98]. Medical nutritional therapy for GDM patients can help control pregnancy weight gain within the normal range, subsequently reducing the risk of obesity in infancy by 73% in their offspring [99]. To date, a variety of dietary interventions have been explored for pregnant women with GDM, including low glycemic index (GI) diets, energy‐restricted diets, adjustments in carbohydrate intake, and modifications to the quality or quantity of fats and proteins. When considering all dietary approaches collectively, modified interventions were significantly linked to a reduction in macrosomia and lower infant birth weight. However, the effects of low‐GI diets on fetal growth remain controversial. For instance, Viana et al. reported that a low‐GI diet significantly decreased birth weight and reduced the frequency of insulin use [100]. Conversely, Wei et al. found that a low‐GI diet, when combined with increased dietary fiber, further reduced the risk of macrosomia compared with a low‐GI diet alone [99]. On the other hand, both Han et al. and Yamamoto et al. found no significant differences in LGA, macrosomia, or birth weight associated with a low‐GI diet. Additionally, energy‐restricted and low‐carbohydrate diets did not show significant differences in fetal growth outcomes. Only the Dietary Approaches to Stop Hypertension (DASH) diet was associated with a lower relative risk of macrosomia; however, it did not appear to influence the risk of LGA [101, 102]. At this stage of research, none of these studies have reported data on long‐term outcomes for the offspring.

#### 3.1.4 The Effect of GDM Mothers' Exercise Status on Obesity in Children's Offspring

Most studies indicate that appropriate physical activity during early and mid‐pregnancy, along with dietary interventions, can help reduce weight gain during pregnancy and decrease the risk of childhood obesity in offspring of mothers with GDM [103]. However, there are currently no established guidelines regarding the optimal duration of exercise. Additionally, some researchers suggest that prenatal exercise may not influence the development of childhood obesity in offspring [104]. This finding could stem from statistical biases in the researchers' data and inadequate adherence to the study protocol. Conversely, some studies explore this phenomenon from a mechanistic perspective. While lifestyle interventions did not lead to significant changes in cord blood gene methylation, they appeared to be associated with a notable attenuation of methylation signatures linked to maternal GDM and the 1‐h and 2‐h plasma glucose (PG) concentrations [105].

#### 3.1.5 The Effect of Sleep in GDM Mothers on Childhood‐Obesity in Their Offspring

The sleep patterns of mothers with GDM significantly affect the weight of their offspring. The study results indicated that an appropriate sleep duration of 7.5 to 8.5 h and good sleep quality influence the incidence of obesity in children. Specifically, sleeping less than 7.5 h was associated with a 1.73‐fold increase in the risk of offspring being overweight or obese. Conversely, sleeping more than 8.5 h increased the risk of overweight/obesity in the offspring by 1.43 times. Moreover, maintaining good sleep quality can reduce the risk of childhood obesity in GDM offspring within the first 24 months. Additionally, ensuring good sleep quality continues to mitigate the risk of childhood obesity in GDM offspring beyond 24 months [106].

#### 3.1.6 The Effect of Folic Acid and Vitamin D Supplementation in GDM Mothers on Childhood‐Obesity in Their Offspring

For the health of both yourself and your offspring, it is important to consume a moderate amount of folic acid along with other essential micronutrients. The current recommendation is to take 400–800 μg/day of folic acid during the first 3 months of pregnancy [107, 108]. Folic acid serves as a crucial coenzyme for the synthesis of nucleic acids and is closely associated with fetal growth and development. Research has indicated that moderate folate intake may act as a protective factor against obesity in the offspring of mothers with GDM. The Boston Birth Cohort Study reported that children whose mothers fell within the lowest quartile of plasma folate had the highest prevalence of overweight or obesity [109]. Similarly, animal studies have demonstrated that excessive folic acid intake can increase the likelihood of obesity in offspring of mothers with GDM [110].

Vitamin D deficiency is common among pregnant women with GDM [111]. In recent years, vitamin D has garnered significant attention for its role in adipose tissue biology and its regulation of human obesity [112]. Studies indicate that low vitamin D levels can lead to T‐cell activation and proliferation, impairing the body's potential to regulate lymphocyte function. It may also promote the production of pro‐inflammatory mediators, thereby sustaining chronic inflammation in adipose tissue [113], while simultaneously regulating glucose metabolism and influencing the synthesis of adipokines in adipose tissue [114]. In animal studies, vitamin D deficiency promotes proliferation and differentiation of preadipocytes and adipocytes in male offspring, leading to obesity [115]. It may also induce obesity by regulating gene expression or modulating levels of parathyroid hormone, calcium, and leptin [116]. A recent meta‐analysis indicates that offspring of mothers with gestational diabetes mellitus (GDM) face a higher risk of overweight with increasing age [117].

### 3.2 Effect of Breastfeeding in GDM Mothers on Childhood‐Obesity in Their Offspring

Breastfeeding in GDM mothers as a protective factor for obesity in offspring. Breast milk is the optimal source of nutrition for healthy growth and development during early childhood [118]. Prolonged breastfeeding for 7 months or longer significantly decreases the likelihood of childhood obesity in normally developing offspring [118]. Similarly, among children born to mothers with GDM, breastfeeding for at least 6 months serves as a protective factor against childhood obesity [119, 120], and may influence growth and development over the subsequent 10 years [121].

Schaefer‐Graf et al. demonstrated that breastfeeding for more than 3 months reduces the risk of childhood overweight by 40% to 50% in the offspring of mothers with GDM [122]. Additionally, breastfeeding for more than 5 months lowers the risk of obesity by 6.7% in children whose mothers had GDM [123], while breastfeeding for over 6 months decreases the prevalence of childhood obesity by 32% in these offspring [124]. Gunderson et al. found that among children of GDM mothers, those breastfed for more than 9 months exhibited lower weight‐for‐length *Z*‐scores and age‐specific weight‐for‐age *Z*‐scores at 1 year of age compared with those breastfed for 0–3 months [125].

The protective effects of breastfeeding on offspring are influenced by various factors, with studies indicating that breastfeeding can provide protection for up to 6 years [126]. However, this benefit may also be affected by the lifestyle of the children. Early exposure to sugar‐sweetened beverages can partially negate the protective effects of breastfeeding [127].

### 3.3 Effect of Gut Microbiota on Obesity in Offspring in GDM Mothers

The first evidence linking the gut microbiota to obesity came from studies on germ‐free mice. Transplanting gut microbes from conventionally housed mice into germ‐free mice increased fat content and insulin resistance levels in the recipients, even with reduced food intake. This demonstrated that gut microbes can enhance fat accumulation in the host [128]. Turnbaugh et al. further confirmed that obese mice exhibited a significantly increased Firmicutes‐to‐Bacteroidetes ratio and demonstrated that their microbiota possessed a greater capacity to extract energy from the diet [129]. Similar phenomena occur in humans. Studies examining dysbiosis in obese patients revealed a substantial reduction in microbial community diversity [130]. Zhu Q et al. found that obesity in infants born to mothers with GDM during infancy is also associated with alterations in gut microbiota [131]. Song Q, Su M, and colleagues analyzed the gut microbiota of infants born to GDM mothers and confirmed that at 6 months of age, these infants exhibited significantly reduced gut microbial diversity. Furthermore, the relative abundance of Lactobacillus and Prevotella—microbial groups negatively correlated with obesity—was diminished [132, 133, 134, 135]. To date, no studies have focused on the late childhood stage of offspring from GDM mothers, leaving this gap awaiting further research.

## 4 Conclusion

The prevalence of obesity is rising globally, coinciding with an aging population. Therefore, the early identification of its risk factors and effective interventions are crucial for the prevention of chronic diseases. Obesity is a recognized risk factor for numerous complications, including type 2 diabetes mellitus, dyslipidemia, hypertension, cardiovascular disease, non‐alcoholic fatty liver disease, and osteoarthritis. Both early onset and prolonged obesity can significantly impact quality of life.

Our study demonstrates that GDM is a significant contributor to obesity in offspring. An intrauterine high‐glucose environment can influence the growth and development of the offspring through the placenta and other pathways. Initially, a high intrauterine glucose level can lead to an abnormal increase in birth weight, which serves as an important indicator for predicting childhood obesity. Over time, this high‐glucose environment can induce changes such as abnormal genome methylation and hyperinsulinemia via placental mechanisms. In later stages, factors such as insulin resistance and increased blood flow to the hypothalamus may persist, leading to long‐term effects.

Consequently, early identification and effective prevention of GDM‐related obesity are vital. Currently, the mechanisms linking GDM to childhood obesity remain unclear and warrant further investigation by researchers in relevant fields. Understanding the occurrence and pathophysiological mechanisms of childhood obesity is essential for identifying, preventing, and treating obesity and its associated diseases. This review aims to provide theoretical innovations for the prevention and treatment of GDM‐related childhood obesity, offer a deeper understanding of the relationship between GDM and the development of childhood obesity in offspring, and inspire new research ideas and directions in the study of obesity and related metabolic diseases.

## Author Contributions

Wantong Chen finished writing the main content of the article, collecting materials and summarizing it, making tables on the basis of observed rules, and drawing images of key mechanisms. Minghui Xi helped drawing images and modified the language content. Shuai Li, Luyi Li, and Danhuai Zhang modified the language content. Ming Gong helped to check the details of the article content. Yueyang Zhao helped to determine the direction of the article and select the journal. Na Wu helped with the discussion, supervised the work, and provided critical feedback. All authors made substantial contributions to conception, took part in drafting the article or revising it critically for important intellectual content; gave final approval of the version to be published; and agree to be accountable for all aspects of the work. All the authors read and approved the final manuscript.

## Funding

This research was supported by the National Natural Science Foundation of China (No. 81700706), the Science Foundation of Liaoning science and technology Department (No. 2023JH2/101700125, No. 2023JH2/101700119) and Liaoning Social Science Planning Fund project (L20BTq003).

## Consent

Agreed to publish.

## Conflicts of Interest

The authors declare no conflicts of interest.

## Data Availability

No datasets were generated or analyzed during the current study.
